# Plasma anandamide and other *N*-acylethanolamines are correlated with their corresponding free fatty acid levels under both fasting and non-fasting conditions in women

**DOI:** 10.1186/1743-7075-7-49

**Published:** 2010-06-14

**Authors:** Michel M Joosten, Michiel GJ Balvers, Kitty CM Verhoeckx, Henk FJ Hendriks, Renger F Witkamp

**Affiliations:** 1 Business Unit Biosciences, TNO Quality of Life, Zeist, The Netherlands; 2 Division of Human Nutrition, Wageningen University, Wageningen, The Netherlands; 3 Business Unit Quality & Safety, TNO Quality of Life, Zeist, The Netherlands

## Abstract

*N*-acylethanolamines (NAEs), such as anandamide (AEA), are a group of endogenous lipids derived from a fatty acid linked to ethanolamine and have a wide range of biological activities, including regulation of metabolism and food intake. We hypothesized that i) NAE plasma levels are associated with levels of total free fatty acids (FFAs) and their precursor fatty acid in fasting and non-fasting conditions and ii) moderate alcohol consumption alters non-fasting NAE levels. In a fasting and non-fasting study we sampled blood for measurements of specific NAEs and FFAs. In the fasting study blood was drawn after an overnight fast in 22 postmenopausal women. In the non-fasting study blood was sampled before and frequently after a standardized lunch with beer or alcohol-free beer in 19 premenopausal women. Fasting AEA levels correlated with total FFAs (r = 0.84; p < 0.001) and arachidonic acid levels (r = 0.42; p < 0.05). Similar results were observed for other NAEs with both total FFAs and their corresponding fatty acid precursors. In addition, AEA (r = 0.66; p < 0.01) and OEA levels (r = 0.49; p <0.02) positively related with BMI. Changes over time in non-fasting AEA levels were correlated with changes in total FFA levels, both after a lunch with beer (r = 0.80; 95% confidence interval: 0.54-0.92) and alcohol-free beer (r = 0.73; 0.41-0.89). Comparable correlations were found for other NAEs, without differences in correlations of each NAE between beer and alcohol free beer with lunch. In conclusion, i) in fasting and non-fasting states circulating anandamide and other N-acylethanolamines were associated with free fatty acid levels and ii) moderate alcohol consumption does not affect non-fasting NAE levels. This suggests that similar physiological stimuli cause the release of plasma *N*-acylethanolamines and free fatty acids in blood. The trials are registered at ClinicalTrials.gov numbers: NCT00524550 and NCT00652405.

## Introduction

**Figure 1:**
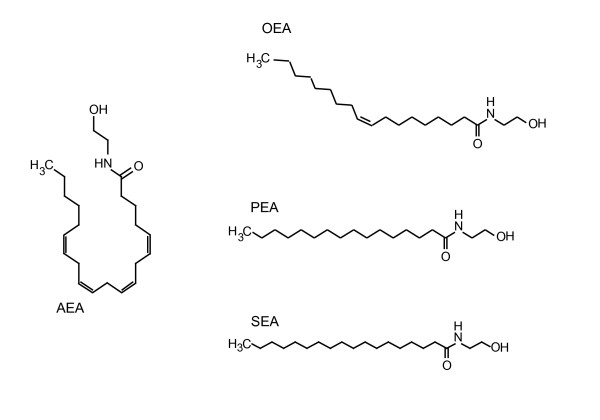
**Structures of the *N*-acylethanolamines: anandamide (AEA), palmitoylethanolamide (PEA), oleoylethanolamide (OEA) and stearoylethanolamide (SEA)**.

*N*-acylethanolamines (NAEs) are a group of lipid mediators, derived from a fatty acid precursor linked to an ethanolamine moiety. The best studied NAE is the endocannabinoid arachidonoylethanolamide (anandamide; AEA). In addition to AEA, NAEs also comprise of other non-endocannabinoids such as palmitoylethanolamide (PEA), oleoylethanolamide (OEA) and stearoylethanolamide (SEA), for which palmitic acid, oleic acid and stearic acid serve as their respective precursor fatty acids (see Figure 1 for structures). NAEs have several biological effects, including regulation of food intake and energy metabolism [1,2].

Several studies have shown that dietary fatty acids determine levels of corresponding NAEs in different tissues, suggesting a link between availability of precursor fatty acids and NAEs formation [3-5]. In obesity, a state characterized by elevated circulating free fatty acids (FFAs), levels of AEA are also increased [6,7]. Conversely, when FFA levels decline after a meal, non-fasting levels of AEA are concomitantly decreased in normal-weight subjects [8]. This made us hypothesize that their release into plasma is regulated by similar physiological stimuli and that plasma NAE levels parallel those of total FFAs and their precursor fatty acids.

Secondly, since AEA and OEA are involved in the regulation of appetite [9], we also wanted to study whether the well-known appetite-inducing effects of acute alcohol consumption [10] might be related to changes in NAEs. Therefore, we investigated the correlation of different NAEs with FFAs in both fasting and non-fasting conditions and the effect of moderate alcohol consumption on non-fasting NAE levels.

## Subjects and Methods

Samples were analyzed from two studies (fasting and non-fasting) conducted at TNO Quality of Life, Zeist, the Netherlands. An independent centralized ethics committee (METOPP; Tilburg, the Netherlands) approved both protocols. Studies are registered at Clinical trials.gov: NCT00524550 and NCT00652405. Eligible women consumed between 5 and 21 units of alcohol per week, were apparently healthy, non-smokers and had no family history of alcoholism. They gave written informed consent.

For the fasting study we used samples from an intervention study completed earlier [11]. At the first day of the intervention subjects arrived at the premises after an overnight fast when blood was drawn.

The non-fasting study was part of a randomized, open label, crossover trial in which subjects consumed two cans of beer (~13 g alcohol each) or two cans of alcohol-free beer (<0.1 g alcohol) (both: Amstel, Amsterdam, the Netherlands) daily for three weeks during dinner. Each three-week intervention period was preceded by a one-week wash-out. At the last day of each treatment, subjects came to the premises for a standardized lunch (1978 kJ; 16.1% protein, 21.0% fat and 62.9% carbohydrate). The two lunches were consumed around noon under similar conditions, 28 days apart. Treatment order (beer vs. alcohol-free beer with lunch) was randomized according to the intervention trial. A first can of study substance was consumed one hour before the start of the lunch, a second can during the lunch. Both cans were consumed within 15 minutes. Blood was sampled before and at several time points after lunch.

In both studies, venous blood was drawn in tubes containing silica as clot activator for serum (FFA analysis) or Potassium Ethylene Diamine Tetra Acid for plasma (NAE analysis). To inactivate fatty acid amide hydrolase (FAAH), phenylmethanesulphonyl fluoride (final concentration: 100 μM) was added to the plasma samples. Total FFA determinations in blood were performed using Olympus analytical equipment and reagents. Plasma levels of NAEs were determined using a LC-MS/MS technique [12]. Specific serum FFA were measured using a high-resolution UHPLC-MS technique [13].

Pearson's coefficient of correlation was calculated to assess correlations between fasting FFA and NAE. In the non-fasting study variables at baseline (t = -60 min before lunch) were compared between treatments with a mixed analysis of variance model. For the correlation between changes over time (non-fasting study), a Fisher's z transformation was applied on individual correlations to correct for deviations from the normal distribution and 95% confidence interval (CI) for each correlation coefficient were calculated [14]. SAS statistical software package (SAS version 9, SAS Institute, Cary, NC) was used to perform statistical analyses. Statistical significance was defined as p < 0.05.

## Results

**Figure 2:**
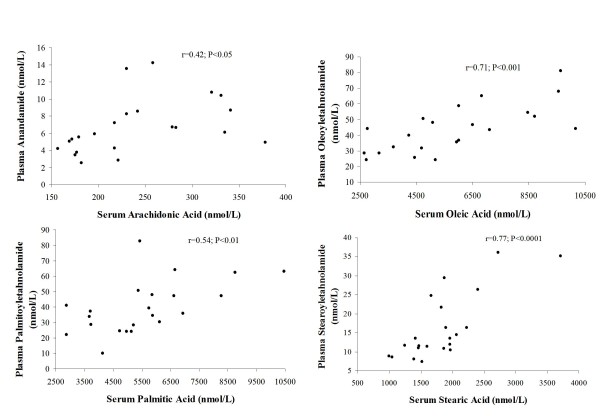
**Pearson correlations between plasma levels of several *N*-acylethanolamines and their corresponding serum levels of fatty acid in twenty-two postmenopausal women after an overnight fast**.

The 22 postmenopausal women enrolled in the fasting study had a mean BMI of 26.3 kg/m^2 ^(range: 19.4-34.1) and a mean age of 55.8 years (range: 51-61). See Table 1 for fasting characteristics. Fasting AEA levels correlated positively with total FFA (r = 0.84, p < 0.001) and with arachidonic acid levels (r = 0.42; p < 0.05). OEA, PEA and SEA levels also positively correlated with serum fasting total FFA levels (All r > 0.44; p < 0.05). Comparable correlations were observed between oleic acid and OEA (r = 0.71; p < 0.001), palmitic acid and PEA (r = 0.54; p < 0.01) and stearic acid and SEA (r = 0.77; p < 0.0001) (Figure 2). Furthermore, AEA (r = 0.66; p < 0.01) and OEA levels (r = 0.49; p < 0.02) were also positively related with BMI.

**Table 1:** Fasting characteristics of the twenty-two postmenopausal women.

	Variable
Glucose (mmol/L)	5.45 ± 0.11
Insulin (pmol/L)	51.9 ± 5.8
Triglycerides (mmol/L)	1.47 ± 0.13
Free fatty acids (mmol/L)	0.59 ± 0.06
Anandamide (nmol/L)	6.8 ± 0.70
Oleoylethanolamide (nmol/L)	43.8 ± 3.3
Palmitoylethanolamide (nmol/L)	40.0 ± 3.7
Stearoylethanolamide (nmol/L)	16.3 ± 1.9

Mean values and standard errors of the mean.

**Figure 3:**
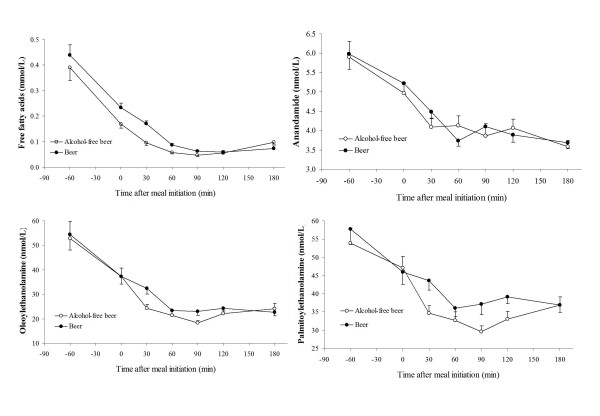
**Means (±SEM) of serum free fatty acid (FFA) concentrations and plasma concentrations of anandamide (AEA), palmitoylethanolamide (PEA) and oleoylethanolamide (OEA) before and during three hours after a lunch with beer (black circles) or alcohol-free beer consumption (open circles) in 19 normal-weight premenopausal women**.

All 19 premenopausal (mean age 23.1 years; range 20-32) women in the non-fasting study had a normal weight (mean BMI: 22.2 kg/m^2^; range: 19.8-24.7). No differences in FFA or NAE levels at baseline were observed between treatments after the three week intervention periods (see Table 2 for fasting characteristics). Curves for AEA, OEA, PEA and total FFA after both meals are shown in Figure 3. Non-fasting changes in total FFA were positively associated with changes in NAEs: (r = 0.73; 95% confidence interval: 0.41-0.89) and (r = 0.80; 0.54-0.92) for AEA; (r = 0.89; 0.73-0.96) and (r = 0.91; 0.78-0.97) for OEA and (r = 0.77; 0.49-0.91) and (r = 0.76; 0.47-0.90) for PEA after a meal either with nonalcoholic beer or beer respectively. Correlations over time of each NAE with total FFAs did not differ between lunches with or without alcohol consumption. In a subgroup of six women, comparable coefficients with NAEs and their specific fatty acids were found for both lunches (data not shown).

**Table 2:** Fasting characteristics after three weeks of consuming beer or alcohol-free beer.

	Alcohol-free beer	Beer	P value
Glucose (mmol/L)	4.97 ± 0.07	5.09 ± 0.07	0.18
Insulin (pmol/L)	44.0 ± 9.2	37.1 ± 9.2	0.55
Triglycerides (mmol/L)	1.17 ± 0.07	1.08 ± 0.07	0.17
Free fatty acids (mmol/L)	0.39 ± 0.04	0.44 ± 0.04	0.18
Anandamide (nmol/L)	5.90 ± 0.39	5.98 ± 0.39	0.86
Oleoylethanolamide (nmol/L)	52.7 ± 6.5	54.4 ± 6.5	0.74
Palmitoylethanolamide (nmol/L)	53.9 ± 4.1	57.8 ± 4.1	0.23
Stearoylethanolamide (nmol/L)	15.1 ± 0.9	15.2 ± 0.9	0.98

Mean values and standard errors of the mean.

## Discussion

The primary findings of this study are i) all fasting and non-fasting plasma NAEs investigated are positively associated with both serum total FFAs and their specific fatty acid precursor and ii) prolonged and acute moderate alcohol consumption does not alter non-fasting NAE levels. These findings imply that circulating NAEs are a reflection of plasma FFA levels.

This suggests AEA levels to be altered in general under conditions characterized by changes in circulating FFAs. Indeed, increased levels of AEA are observed in obesity, diabetes, and eating disorders such as anorexia and binge-eating disorder [6,8,15]. Furthermore, increased levels of circulating AEA, OEA and PEA were found in patients with liver cirrhosis [16]. Besides the correlation with fasting FFAs, we also observed a strong correlation between AEA and OEA with BMI in postmenopausal women. This is in line with previous work which observed higher levels of AEA among obese postmenopausal women compared with normal-weight counterparts [6].

Although the release of NAEs into plasma may still be biologically relevant, it remains to be determined of the observed relation between FFA and NAE levels is also causal. Perhaps, the increase in FFA levels is paralleled by increased membrane phospholipid cleavage. The increased levels of AEA and 2-arachidonoylglycerol (2-AG), another arachidonic acid-derived endocannabinoid, have led to the concept of the 'overactivated' endocannabinoid system in obesity in which FAAH expression is reduced [6,7,17]. However, our data suggest that increased levels of endocannabinoids and other NAEs in obesity could take place in parallel with increased FFA concentrations and does not necessarily reflect a functional change of the endocannabinoid system alone.

Only one other study investigated non-fasting AEA concentrations in humans, without reporting results on FFAs [8]. They found decreased AEA levels one hour after a lunch. Possibly the physiological stimuli involved in the decrease of FFA levels after consumption of a meal also contribute to a reduced release of NAEs from membrane phospholipids. However, a decreased activity of enzymes involved in NAE synthesis such as N-acylphosphatidylethanolamine hydrolyzing phospholipase (NAPE-PLD) or increased FAAH activity can not be excluded.

To our best knowledge, this is the first human study in which NAEs are reported after both prolonged and acute moderate alcohol consumption. Acute alcohol consumption is generally known to stimulate appetite [10]. In our study, circulating non-fasting NAEs did not differ between a lunch with or without alcohol consumption. It thus seems unlikely that the acute appetite-inducing effects of alcohol are caused by alterations in circulating NAEs.

The observed correlations between NAEs and FFAs are persistent for several NAEs in various populations (pre- and postmenopausal women) and under different conditions (fasting and non-fasting). However, some limitations warrant consideration. It remains to be established whether changes in plasma NAEs will also modify NAEs in target tissues such as adipose tissue, brain, liver and intestines as seen in animal studies [3] and to which extend these changes affect physiology. The exact site of NAE synthesis is unknown, but the liver, adipose tissue or blood cells could contribute to the changes in NAE levels. Furthermore, other regulators such as insulin may affect NAE levels [18]. Finally, we did not measure 2-AG, another important endocannabinoid, or other NAEs.

In conclusion, we provide evidence that in humans fasting peripheral NAEs are positively correlated with both serum total fasting FFA and their specific fatty acid precursor. Furthermore, we showed that non-fasting changes over time in AEA and related NAEs are positively correlated with non-fasting changes in free fatty acids, independent of alcohol consumption. This suggests that circulating N-acylethanolamines might be a reflection of free fatty acids in blood. The biological significance of these findings is still unknown and requires further investigation.

## Competing interests

The authors declare that they have no competing interests.

## Authors' contributions

MJ designed study protocols, performed statistical analysis and drafted the manuscript. MB developed the NAE analysis technique, participated in NAE and FFA analysis and drafted the manuscript. KV developed the NAE analysis technique was involved in NAE analyses. HH and RW participated in the design and coordination of the study and significantly helped drafting the manuscript. All authors read and approved the final manuscript.
